# Basal cell adenocarcinoma of a hard palate minor salivary gland: case report and review of the literature

**DOI:** 10.1186/1758-3284-1-41

**Published:** 2009-12-23

**Authors:** Bryan K Ward, Raja R Seethala, E Leon Barnes, Stephen Y Lai

**Affiliations:** 1 Department of Otolaryngology, Johns Hopkins University School of Medicine, Baltimore, MD, USA; 2 Department of Pathology, University of Pittsburgh School of Medicine, Pittsburgh, PA, USA; 3 Department of Head and Neck Surgery, University of Texas MD Anderson Cancer Center, Houston, TX, USA; 4 Department of Molecular and Cellular Oncology, University of Texas MD Anderson Cancer Center, Houston, TX, USA

## Abstract

**Objective:**

Basal cell adenocarcinoma of a minor salivary gland is extremely rare. The goal of this report is to increase awareness of this rare disease and to review and discuss the differential diagnosis and important considerations in treatment.

**Study Design:**

Case report and review of the literature.

**Methods:**

Case report of a basal cell adenocarcinoma of a hard palate minor salivary gland and review of the literature of basal cell adenocarcinoma.

**Results:**

Basal cell adenocarcinomas are slow-growing tumours that most commonly involve the parotid gland and very rarely involve minor salivary glands. Although recurrence rates for these tumours are high, mortality rates are low. Histological diagnosis is important to distinguish this tumour from adenoid cystic carcinoma given the significant difference in disease prognosis.

**Conclusions:**

Diagnosis of these tumours must be made histologically. Recommended treatment options include wide local excision with radiotherapy reserved for close surgical margins or for local recurrence.

## Background

Minor salivary gland neoplasms constitute up to approximately 25% of all salivary gland tumours [1,2]. The incidence of malignancy of these tumours is slightly greater than half but varies by series. Basal cell adenocarcinoma (BCAC) is a rare salivary gland malignancy that occurs most commonly in the parotid gland [3]. In this report we describe a case of an older woman presenting with basal cell adenocarcinoma of a hard palate minor salivary gland.

## Case Presentation

**Figure 1:**
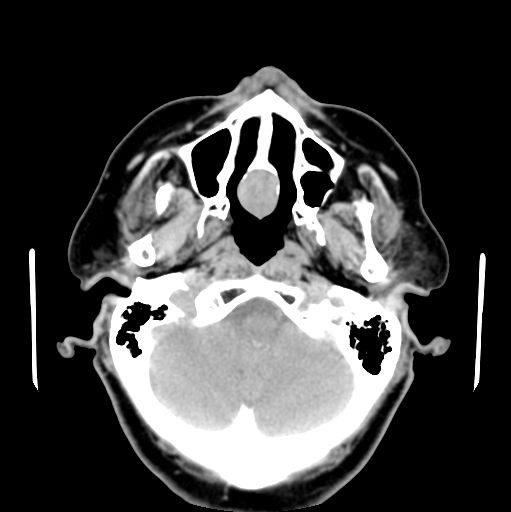
**Axial non-contrast computed tomography scan demonstrating mass at posterior septum with extension into hard palate**.

A 69 year-old woman presented with a one-year history of a persistent ulcer on her hard palate and a poorly fitting upper denture plate. During this period, the patient had no weight loss, symptoms of nasal obstruction, nasal discharge, or dysesthesias. A midline 2 × 2.5 cm mass was present on her central hard palate with raised surfaces, an area of central ulceration, and soft palate extension. No lymphadenopathy was detected. An axial computed tomography (CT) scan image revealed a 2-cm heterogeneously enhancing mass at the nasal septum extending into the hard palate (Figure 1) and biopsy of the mass indicated possible carcinoma.

**Figure 2:**
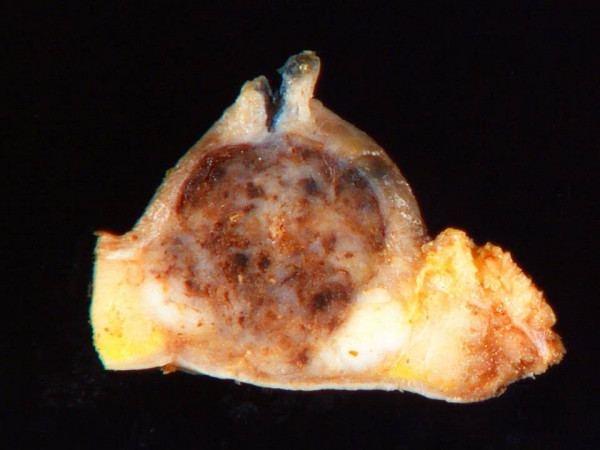
**Bisected gross pathologic specimen with areas of focal hemorrhage**.

**Figure 3:**
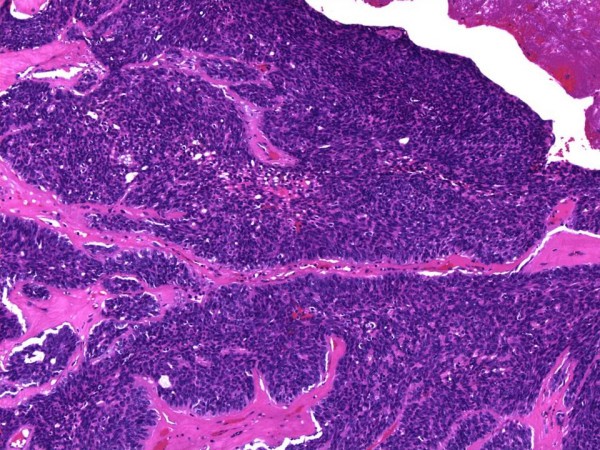
**Histologic appearance of solid/trabecular nests of cells, scant cytoplasm, vesicular nuclei and peripheral palisading**. Hematoxylin-eosin, magnification ×100.

**Figure 4:**
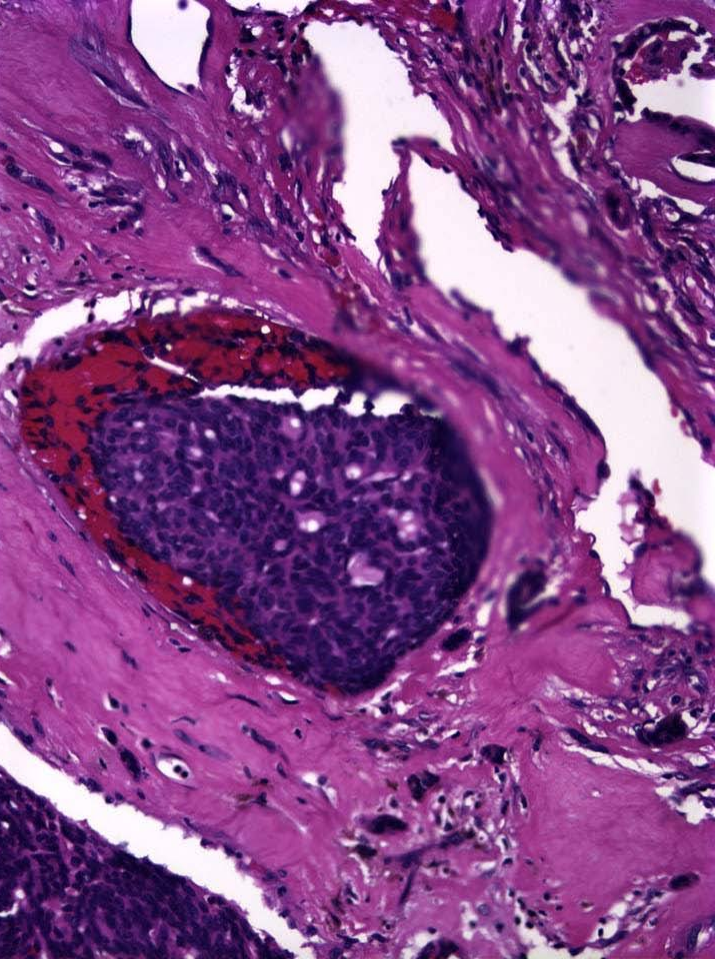
**Histologic appearance of angiolymphatic invasion**. Hematoxylin-eosin, magnification ×300.

The patient was taken to the operating room for a combined transnasal endoscopic septectomy and a transoral maxillectomy. Septal incisions were made around the mass and carried down to the maxillary crest. The mouth was opened, and a McIvor mouth gag was placed to maintain maximal exposure. Wide local excision of the mass included a portion of the anterior soft palate and the oral incision was connected to the septal incision to permit en bloc resection. On gross pathologic examination, the tumour had a cystic hemorrhagic granular tan brown appearance and bone invasion (Figure 2). Low power examination (Figure 3) demonstrated solid to trabecular nests of tumour cells with scant cytoplasm, vesicular nuclei and peripheral palisading. At the periphery, a focus of angiolymphatic invasion was identified (Figure 4). The final pathology diagnosis was BCAC of a hard palate minor salivary gland. Neither radiotherapy nor neck dissection was pursued. The patient has remained without recurrence or disease progression for the 12 months since her operation. Written consent for publication was obtained from the patient.

## Discussion

BCAC typically arise in adults older than 60 years and have no gender predominance [4]. Patients present with a slow-growing mass that unlike this case, usually presents without ulceration [1]. Local pain is present in about 25% of patients. The 2005 World Health Organization classification categorizes BCAC as a low-grade tumour with a favourable prognosis [5]. These tumours were first recognized in 1978 and account for approximately 1.6% of all salivary gland neoplasms [3]. The vast majority occur in the parotid gland (about 90%) [6-8], followed by the submandibular gland and minor salivary glands [1].

BCAC is believed to arise from pluripotent ductal reserve cells. Grossly, these tumours appear solid grey-tan and grow as large as 7 cm in diameter [9]. BCAC has four major histologic growth patterns: solid, tubulotrabecular, cribriform and membranous. The solid pattern is the most common and the most likely to present with perineural invasion, though the prognostic significance of perineural invasion in BCAC is unknown. The membranous type has an appearance resembling dermal eccrine cylindromas. A syndrome of numerous basal cell adenomas, dermal cell cylindromas, and trichoepitheliomas has been described called Brooke-Spiegler Syndrome [4,9]. Most cases of BCAC are believed to develop de novo but as many as 25% of cases may arise from a pre-existing basal cell adenoma [10,11].

The major pathologic differential diagnostic considerations for BCAC are basal cell adenoma and adenoid cystic carcinoma. As its malignant counterpart, BCAC shares many histologic characteristics with basal cell adenoma but is distinguished from basal cell adenomas often only by invasion of local structures or by perineural or angiolymphatic invasion [12]. Distinguishing BCAC from adenoid cystic carcinoma is important due to the poorer prognosis and higher prevalence of the latter disease. Major distinguishing features of adenoid cystic carcinoma are the presence of dark hyperchromatic angulated nuclei which are in contrast with the vesicular nuclei of BCAC. Additionally BCAC often have prominent peripheral palisading of the outer layer that adenoid cystic carcinomas lack [9]. Immunostains are of uncertain value in this differential.

BCAC of a minor salivary gland is an extremely rare tumour with 22 reported cases in the literature. In recent series' of minor salivary gland tumours, BCAC account for <1% of all minor salivary gland tumours [12-16]. Overall, minor salivary gland tumours typically are located on the hard palate [16]; however, in the few available cases of minor salivary gland BCAC, they arise commonly from the buccal mucosa (9) in addition to the palate (7), followed by lip and tongue [17]. Patients present with a slow-growing mass in an area of minor salivary gland distribution. The prognosis of BCAC of a minor salivary gland appears similar to that of BCAC of other salivary glands. The treatment of choice is wide local excision. Since these tumours seldom metastasise to cervical lymph nodes, routine neck dissection is not recommended. The mortality rate for this tumour is also low, although reported local recurrence rates are high. Parashar et al. [17] noted that of 17 patients with BCAC of minor salivary glands with follow-up between 2 and 14 years, 2 patients died of the disease, both within 4 years. As with minor salivary gland malignancies in general, postoperative radiation is recommended for close surgical margins or following surgical excision of recurrent disease.

## Conclusions

Basal cell adenocarcinoma (BCAC) is a rare salivary gland tumour that carries a favourable prognosis. Although there are few reported cases of BCAC of minor salivary gland origin, BCAC should remain a diagnostic consideration. Since these tumours typically require only local excision, they must be distinguished histologically from adenoid cystic carcinoma, which has a poorer prognosis and often necessitates a more aggressive clinical approach.

## Consent

Written informed consent was obtained from the patient for publication of this case report and any accompanying images. A copy of the written consent is available for review by the Editor-in-Chief of this journal.

## Competing interests

The authors declare that they have no competing interests.

## Authors' contributions

BKW performed the literature research, participated in the clinical care of the patient and drafted the manuscript. RRS and ELB participated in drafting the manuscript and in the patient's diagnosis. SYL performed the patient's surgery and participated in drafting the manuscript. All authors read and approved the final manuscript.
